# Prevalence of fibromyalgia in France: a multi-step study research combining national screening and clinical confirmation: The DEFI study (Determination of Epidemiology of FIbromyalgia)

**DOI:** 10.1186/1471-2474-12-224

**Published:** 2011-10-07

**Authors:** Serge Perrot, Eric Vicaut, Dominique Servant, Philippe Ravaud

**Affiliations:** 1 Service de Médecine Interne et Thérapeutique, Hôtel Dieu, Université Paris Descartes, INSERM U 987, 1 Place du Parvis Notre Dame, 75004 Paris, France; 2 Unité de Recherche Clinique, Hôpital Lariboisière, 2 Rue Ambroise Paré, 75010 Paris, France; 3 Unité Stress et Anxiété, Hôpital Fontan, CHRU de Lille, 6 Rue André Verhaeghe 59037 Lille Cedex, France; 4 Département d'épidémiologie, Hôtel Dieu, Université Paris Descartes, 1 Place du Parvis Notre Dame, 75004 Paris, France

## Abstract

**Background:**

Fibromyalgia is a common disease, but little is known on its real prevalence in France. This epidemiological study aimed to assess fibromyalgia (FM) prevalence in the French metropolitan population, based on a multi-step sampling analysis, combining national screening and clinical confirmation by trained specialists.

**Methods:**

a sampling method on the entire national territory was used: patients over 18 years of age accepting to take part in the study were contacted by telephone using the LFES Questionnaire, a screening test for FM. The, for patients detected by the LFESQ, a visit with a FM-trained rheumatologist was proposed to confirm FM, based on 1990 ACR criteria. Each detected patient completed the following self-questionnaires: SF36, HADS, stress VAS, Co-morbidities and Regional pain score.

**Results:**

3081 patients were contacted in 5 representative French regions, of which 232 patients were screened for FM. A fibromyalgia diagnosis was then confirmed by rheumatologist in 20 cases (17 female and 3 male, 56.9 ± 13.2 years). The final estimated FM prevalence was 1.6 (CI95: 1.2%; 2.0%). No significant difference was detected between the patients accepting (CS+) and refusing (CS-) rheumatologist visit for the SF36 score, regional pain score, stress VAS scale and co-morbidities. In patients detected for FM by the LFESQ, we found a statistically significant decrease in quality of life and a statistically significant increase in stress level in patients with a confirmed diagnosis (FM+) (6.3 ± 1.9) compared to patients with an invalidated diagnosis (FM-) (4.4 ± 2.8; p = 0.007). The study also demonstrated a significant association, independently of ACR criteria, between the diagnosis of FM and several factors such as regional pain score > 10, elevated stress level, low SF36 scale score and presence of gastro-intestinal disorder co-morbidities.

**Conclusion:**

Fibromyalgia is a common condition; the 1.6% prevalence calculated in the French population in our study corroborates the figures published in the European literature. Our results also suggest that criteria such as regional pain score, stress level or SF36 quality of life, could represent useful tools in fibromyalgia diagnosis.

## Background

Fibromyalgia (FM) is a debilitating chronic disease characterized by pain described by patients as muscular and/or skeletal, diffuse and chronic, exacerbated by pressure from some tendon insertion points associated with severe long-term fatigue. For many years, FM diagnosis was based on the 1990 American College of Rheumatology (ACR) classification criteria [1], initially not dedicated to the diagnosis. New diagnosis criteria [2] and screening tool [3] have been proposed in 2010. Progress in imaging (MRI and PET) [4] and neurobiology in the last 10 years [5] have enabled a better understanding of the physiopathology of this disease which appears in association with a central pain modulation disorder characterized by nociceptive and neuropathic pain pathway dysfunction. Affected patients display a lowering of the pain perception threshold, with the induction of pain by stimuli not normally inducing pain (allodynia). Guidelines for FM management have been published by the European League Against Fibromyalgia (EULAR) [6]. In epidemiological terms, FM is not a rare disease and its estimated frequency varies according to the population and the methodology. Its prevalence is reported to be 2 to 6% in the case of patients attending general practitioners, 5 to 8% in hospitalized patients and 14 to 20% in rheumatology consultations [7-9]. In France, very few studies have been conducted [10] and accurate prevalence figures for FM are not available, prevalence figures between 0.5% and 5% have been suggested. The aim of this study was to assess the prevalence of fibromyalgia in the general population in France, with a multi-step study, combining large national screening process and confirmed diagnosis by trained specialists.

## Methods

The study in question was a multi-centre, national, cross sectional interventional study.

### General study design

A probabilistic sample of 6000 households, selected at random from the telephone directory, was prepared in the general population in 5 geographic representative regions in France: the conurbations of Lille, Grenoble, Toulouse and the Val de Marne and Ille-et-Vilaine *départements*. Each region was represented by 1200 households defined as "regular households" (primary residence and at least one member aged 18 years or over). Ethical approval was obtained from the Ethical Committee Paris Ile de France, and from the CNIL for data analyses.

### Selection of patients with potential or established fibromyalgia

A single member of each household was contacted by the IPSOS, a market-research company specialized in poll studies, by telephone on the basis of the following criteria: subject aged 18 years of over, accepting to take part in the study and capable of understanding and answering the questions. A questionnaire was completed with the following data: total number of adult members of the household, specifying the number of females and males, demographic characteristics of the interviewee (gender, month and year of birth, socio-professional group). To determine whether the subject had fibromyalgia or not, the "London Fibromyalgia Epidemiology Study Screening Questionnaire" or LFES-SQ [11], validated in its French version (10), was used for the telephone interviews. A subject was deemed to have potential or established fibromyalgia if they gave a positive response to the 4 pain criteria alone (In the past 3 months: 1. Have you had pain in muscles, bones, or joints, lasting at least 1 week? 2. Have you had pain in your shoulders, arms, or hands? On which side? Right, left, or both? 3. Have you had pain in your legs or feet? On which side? Right, left, or both? 4. Have you had pain in your neck, chest, or back?) or to the 4 pain criteria plus the 2 criteria relating to fatigue (1. Over the past 3 months, do you often felt tired or fatigued? 2. Does tiredness or fatigue significantly limit your activities?)

### Follow-up of patients with potential or established fibromyalgia

In total, 24 rheumatologists (4 to 5 per region) were selected to take part in the study. To ensure a homogeneous diagnosis, the rheumatologists received an extensive and standardized training on chronic pain, fibromyalgia diagnosis, and the handling of the various questionnaires. Patients with potential or established fibromyalgia selected following the LFES-SQ questionnaire were offered a visit with a rheumatologist to confirm the fibromyalgia diagnosis. Patients who refused this consultation had the option of receiving, by mail, questionnaires to assess the condition, the regional pain score and questionnaires to assess some impact of the condition: quality of life (QoL) questionnaire by SF36, as a generic QoL questionnaire [12], emotional impact scale by HADs (Hospital Axiety and Depression scale) to detect anxiety and depression [13], current stress level measured using the a 100 mm Visual Analog Scale (VAS), presence of Co-morbidities and regional pain score. They were asked to send back the questionnaires via postal mail.

### Diagnosis and care provided by rheumatologist

During the consultation, the rheumatologist drew up an inventory of the patient's profile, his/her rheumatology or other medical history, demographic characteristics and socio-professional status. Screening for pain sites was performed according to the 1990 ACR criteria (1) for FM diagnosis. A questionnaire was completed by the rheumatologist with the following information: patient's demographic data, history of chronic pain, previous FM history, patient's previous rheumatological history, patient's other major medical history, ACR1990 FM criteria. The patient also completed the following questionnaires, with assistance from the rheumatologist: SF36, HADS, stress level assessed using the VAS scale, co-morbidities and regional pain score.

#### Calculation of prevalence and statistical analysis

An estimation of the FM prevalence in the interviewed population was calculated with its 95% confidence interval using the formula below where Ndiagnosis is the number of FM cases confirmed by the rheumatologist and Nrefusing-consultation the number of cases of FM estimated in the population of patients refusing the consultation.

$$\text{Prevalence (\%)}=\frac{{\text{N}}_{\text{diagnosis}}+\left(\frac{{\text{N}}_{\text{diagnosis}}}{{\text{N}}_{\text{accepting consultation}}}\times {\text{N}}_{\text{refusing consultation}}\right)}{{\text{N}}_{\text{interview}}}\times \text{10}0$$

The statistical analysis was conducted in SAS version 9.1.3 in Windows XP. Descriptive statistical analyses were conducted for the quantitative variables: mean, standard deviation, median, minimum and maximum and 95% confidence interval (95% CI) and for the qualitative variables (frequencies, percentages and 95% confidence interval).

## Results

**Figure 1:**
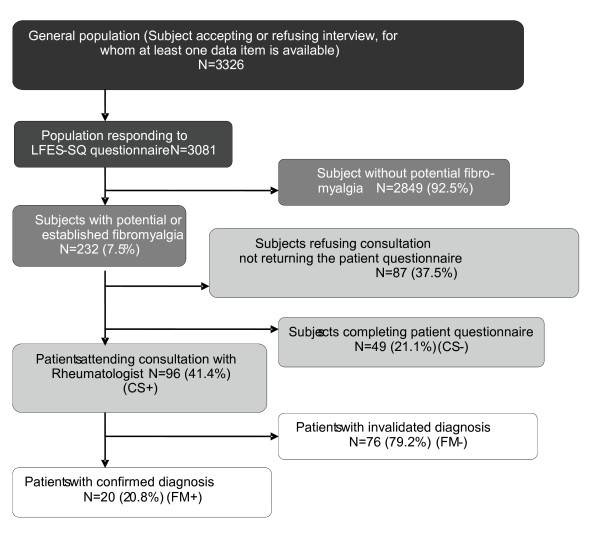
**Patient distribution in the study**.

Between April 21, 2008 and September 29, 2008, 6000 households were contacted by telephone, 3326 subjects accepted to take part in the study and were contacted, of whom, 245 refused or withdrew during the questionnaire. In the end, 3081 subjects responded fully to the telephone questionnaire (LFES-SQ). Following this selection, 232 (7.5%) subjects were classified as having potential or established fibromyalgia (LFES-SQ+). Of the 232 LFES-SQ+ patients, 96 (41.4%) attended a rheumatology consultation (CS+), 49 (n = 21.1%) returned the patient questionnaires without attending the consultation (CS-) and 87 (37.5%) did not attend the consultation or complete the questionnaires. The patient distribution in the study is described in Figure 1.

### Confirmation of fibromyalgia diagnosis

Of the 96 CS+ patients, a fibromyalgia diagnosis was confirmed clinically by the rheumatologist in 20 patients (FM+) based on ACR criteria (presence of the three following criteria: history of diffuse pain, chronic pain progressing for more than 3 months and pain on digital palpation of 11 to 18 points) and invalidated in 76 patients (FM-). The latter did not meet all the ACR criteria. We did not found any patient with a previous diagnosis of FM.

### Prevalence of fibromyalgia in the study population

Of the 232 patients detected with potential or established fibromyalgia, 20 cases were confirmed by the rheumatologist. Considering that the prevalence of FM was identical in both groups of patients accepting and rejecting consultation, the estimated number of cases of FM in the CS- patients would be 28 cases, as indicated in Table 1, bringing the total estimated number of cases of FM in the interviewed population to 48. The estimated prevalence of FM in the interviewed population is thus 1.6% (95%CI:1.2%; 2.0%). This prevalence varied according to the regions; 0.8% (0.1%; 1.5%) in Toulouse, 0.8% (0.2%; 1.5%) in Grenoble, 1.7% (0.7%; 2.7%) in Lille, 1.9% (0.9%; 2.9%) in Ille-et-Vilaine and 2.7% (1.3%; 4.1%) in Val-de-Marne.

**Table 1:** Number of confirmed and estimated cases of fibromyalgia in interviewed population

A	B	C	D	E (B-C)x D/C	F (D+E)
Interviewed patients	Patients with potential or established fibromyalgia	Patients accepting consultation	Number of cases of FM confirmed by rheumatologist	Number of estimated cases of FM in patients refusing consultation	Total number of cases of FM
	(LFES-SQ+)	(CS+)	(FM+)	(CS-)	

3081	232	96	20	28	48

LFES-SQ: London Fibromyalgia Epidemiology Study- Screening Questionnaire, FM: Fibromylagia, CS: Consultation?

### Characteristics of the study population

Table 2 describes the demographic characteristics of the main populations analyzed. The interviewed population (n = 3081) consisted of 61% women. Taking the patients responding to the LFES-SQ questionnaire into account, the LFSE-SQ+ patient group consisted of a majority of women (71.1% vs 60.3% p = 0.001) and older subjects compared to the LFESSQ- patient group (61.8 ± 17.3 years vs 54.4 ± 19.2 years, p < 0.0001). The patient population accepting the consultation (CS+) consisted of a majority of women (69.8%), the mean age was 58.4 ± 14.6 years and 55.9% of those stated they had an occupation. The time elapsed since the first chronic pain in CS+ patients was, on average, 12.7 ± 11.6 years. No significant difference was observed for the demographic data between the FM+ and FM- patients. Table 2 describes the co-morbidities in CS+ and CS- patient and also in FM+ and FM- patients. No significant difference was observed with the exception of the more frequent previous history of rheumatoid arthritis in the CS- group (19.2% vs 7.4% p = 0.037) and statistically more frequent cramp-type digestive disorders in the FM+ patients compared to the FM- patients (79.0%; 95%CI: 60.6; 97.3) vs (48.0%; 95%CI: 36.5; 59.4, p = 0.016).

**Table 2:** Demographic characteristics of populations analyzed

	Patients responding to LFES-SQ questionnaire		Patients with potential or established fibromyalgia		Patients attending consultation with rheumatologist	
	LFES-SQ+*	**LFES-SQ-**	**CS+**	**CS-**	**FM +**	**FM -**

Number of patients	232	2849	96	49	20	76

Age; Mean ± SD 95%CI	**61.8 ± 17.3** [59.5;64.0]	**54.4 ± 19.2** [53.7;55.1]	58.2 ± 14.7 [55.2;61.2]	62.1 ± 18.6 [56.7;67.4]	56.9 ± 13.2 [50.7;63.1]	58.8 ± 15.0 [55.4;62.3]

Males N (%) 95%CI	67 (28. 9) [23.1;34.71]	1132 (39.7) [37.9;41.5]	28 (29.2) [20.1;38.3]	16 (32.7) [19.5;45.8]	3 (15.0) [0.0;30.7]	26 (34.2) [23.5;44.9]
Females N (%) 95%CI	**165 (71.1) **[65.3;77.0]	**1717 (60.3) **[58.5;62.1]	68 (70.8) [61.7;79.9]	33 (67.4) [54.2;80.5]	17 (85.0) [69.4;100.0]	50 (65.8) [55.1;76.5]

LFES-SQ = London Fibromyalgia Epidemiology Study- Screening Questionnaire; CS+: patients attending consultation with rheumatologist and completing questionnaire; CS-: patients completing questionnaire but not attending consultation with rheumatologist; FM+: = confirmed diagnosis of fibromyalgia; FM-: = non-confirmed diagnosis of fibromyalgia

### Analysis of patient questionnaires (SF36, regional pain score and stress level assessed with VAS, anxiety and depression)

The scores of each of the eight SF-36 scales, and the psychological and physical summary scores were standardized to obtain a score of 0 to 100 where 50 (±10) corresponds to the 1998 reference U.S. population [14]. The results of the harmonized SF36 scores, co-morbidities, the regional pain score and the stress level VAS scale were compared between the CS+ (n = 96) and CS- (n = 49) with potential or established fibromyalgia and also the FM+ (n = 20) and FM- (n = 76) patients.

**Figure 2:**
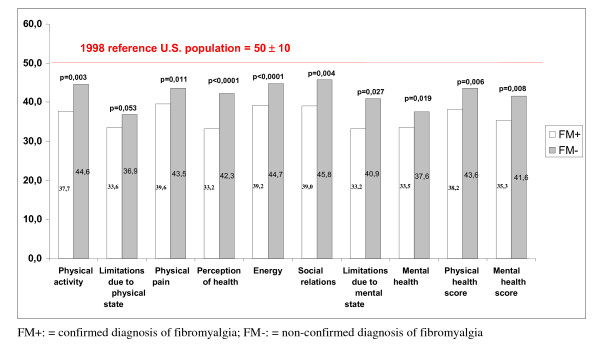
**Comparison of mean SF36 scores between patients with confirmed (FM+) and non-confirmed (FM-) fibromyalgia diagnosis**.

For the group of patients with potential or established fibromyalgia (n = 232), the results did not demonstrate any significant difference for the SF36 score, RPS and stress VAS scale. On the other hand, a statistically significant decrease in the quality of life of the FM+ patient group was observed compared to the FM- patient group on 7 of the 8 scores in the SF36 scale (Figure 2). The mean stress level measured using a visual analog scale was statistically higher in the FM+ patients compared to the FM- patients (6.3 ± 1.9: 95%:5.4; 7.1) vs (4.4 ± 2.8 :95%CI: 3.8; 5.1, p = 0.007). Patients were considered as having an anxious disorder or a depressive disorder if the HADs subscore was higher than 10, and these are the patients mentionned in the table 3.

**Table 3:** Co-morbidities in CS+/CS- and FM+/FM- patient

	Patients with potential or established fibromyalgia		Patients attending consultation with rheumatologist	
	**CS+**	**CS-**	**FM+**	**FM-**

**Number of patients**	**96**	**49**	**20**	**76**

Co-morbidities (%)				
Long-term fatigue [95% CI]	76.3 [67.7; 85.0]	68.9 [55.4; 82.4]	85.0 [69.4; 100.0]	74.0 [63.9; 84.0]
Cramp type digestive disorders [95% CI]	54.4 [44.2; 64.5]	52.4 [37.3; 67.5]	**79.0** [60.6; 97.3]	**48.0** [36.5; 59.4]
Headaches [95% CI]	53.4 [43.0; 63.8]	41.0 [25.6;56.5]	66.7 [44.9; 88.4]	50.0 [38.3; 61.7]
Anxiety 95% CI]	75.8 [67.2; 84.4]	74.4 [61.4; 87.5]	84.2 [67.8; 100.0]	73.7 [63.8; 83.6]
Depression [95% CI]	39.3 [29.2; 49.5]	40.5 [24.7;56.4]	47.4 [24.9; 69.8]	37.1 [25.8;48.5]
Rheumatoid arthritis [95% CI]	**7.4** [2.1; 12.6]	**19.2** [7.9; 30.4]	15.0 [0.0; 30.7]	5.3 [0.3; 10.4]
Systemic lupus erythematosus [95% CI]	1.1 [0.0; 3.2]	0.0 [0.0; 0.0]	0.0 [0.0; 0.0]	1.4 [0.0; 4.0]

CS+: patients attending consultation with rheumatologist and completing patient questionnaire; CS-: patients completing patient questionnaire without attending consultation with rheumatologist; FM+: = confirmed diagnosis of fibromyalgia by a rheumatologist; FM-: = non-confirmed diagnosis of fibromyalgia by a rheumatologist

The maximum values of the Regional Pain Score ranged from 3.0 to 19.0 and a statistically significant difference was also observed between the FM+ and FM- patient groups, with respect to the mean regional pain score (13.2 ± 4.3 vs 9.0 ± 4.4, p < 0.001), as indicated in Table 3.

### Factors associated with confirmation of diagnosis by the rheumatologist

**Figure 3:**
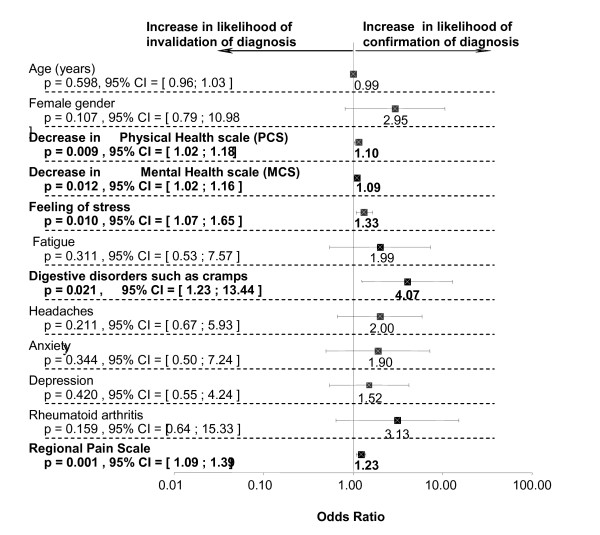
**Risk Factors associated with fibromyalgia diagnosis**.

A search for factors associated with an increase in the likelihood of diagnosis of FM by the rheumatologist was conducted in the patient population attending the consultation (N = 96). The following factors were included in the univariate analysis; gender, SF36, stress level VAS, co-morbidities (long-term fatigue, cramp type digestive disorders, headaches, anxiety, rheumatoid arthritis, systemic lupus erythematosus), and the regional pain score. The results are summarized in figure 3 and demonstrate that the likelihood of having an increase in the diagnosis of fibromyalgia by the rheumatologist was statistically linked with increases in the stress level assessed on the VAS (p = 0.010), the presence of cramp type digestive disorders (p = 0.021), a decrease in some quality of life scores such as the physical health (p = 0.009) and mental health (MCS) (p = 0.012) scale and an increase in the regional pain score (p < 0.001). A correlation study between the regional pain score and the confirmation of diagnosis by the rheumatologist was conducted and demonstrated that the best sensitivity/specificity ratio was obtained for a regional pain score threshold equal to 10 with a 75% sensitivity and a 67.1% specificity (Table 4).

**Table 4:** Regional pain score

	Patients with potential or established fibromyalgia		Patients attending visit with rheumatologist	
	**CS+**	**CS-**	**FM+**	**FM-**

**Number of patients**	**96**	**49**	**20**	**76**

Regional Pain Score				
Mean ± Standard Deviation (SD) [95% CI]	9.8 ± 4.7 [8.9; 10.8]	8.5 ± 4.7 [7. 2; 10.0]	**13.2 ± 4.3** [11.2;15.2]	**9.0 ± 4.4** [8.0; 9.0]

CS+: patients attending consultation with rheumatologist and completing questionnaire; CS-: patients completing questionnaire but not attending consultation with rheumatologist; FM+: = confirmed diagnosis of fibromyalgia; FM-: = non-confirmed diagnosis of fibromyalgia

### Limitations of the study

Our study has some limitations, related to the methodology: patients were contacted by telephone, and the LFESQ was used by telephone interviews to screen the patients. Patients that did not accept to attend a visit to a rheumatolgist were asked to fill-in questionnaires, and to send it back by postal mail. All these steps were performed without physician.

## Discussion

To assess the FM prevalence in the French population, this epidemiological study was conducted based on the same methodology as that described by White KP et al [15] in the "London Fibromyalgia Epidemiology Study". In our study, this methodological approach validated by the scientific community was used to eliminate regional bias for the screening of patients with potential fibromyalgia and homogenize the fibromyalgia diagnosis in patients screened using this method. The screening for FM patients was conducted in 2 phases; screening of potential patients in a representative sample of the general population using the LFES-SQ questionnaire and confirmation of cases of established FM by a rheumatologist, using ACR 1990 FM criteria. The medical investigators involved in the study were not required to be representative since the representation of the study population was ensured by the sampling of the subjects themselves. The choice of rheumatologists was preferred for medical demographic reasons and because rheumatologists are familiar with this condition. This was the first study conducted in France combining telephone screening and confirmation of the diagnosis based on ACR 1990 FM criteria. Thus, accounting for the LFES-SQ questionnaire alone, the prevalence of FM amounted to 7.5% in the interviewed population. This prevalence was estimated at 1.6% (95%CI:1.2%; 2.0%) when FM was diagnosed by the rheumatologist based on ACR 1990 FM criteria. A number of FM prevalence studies have been conducted and have demonstrated great variability in prevalence figures between 0.5% and 57% [6], [15-20] according to the countries, criteria and data collection methods used. In practice, it is difficult to compare these results, firstly, due to the lack of homogeneity in the populations studied and, secondly, due to the difference in the methodologies used. Nevertheless, the prevalence of 1.6% measured in this study corroborates the prevalence figures of 2.2% (1.3%; 3.1%) published by Bannwarth et al [10] with another screening methodology, without diagnosis confirmation. North American studies [15] using the same methodology have reported FM prevalence figures in adults not staying in healthcare establishments of 2% to 3.3% [6,15]. The lower prevalence rate observed in our study could be explained by the small number of patients accepting the visit with the rheumatologist and by the strict application of ACR 1990 FM criteria which would explain the small number of cases of FM confirmed by the rheumatologists in our study. Indeed, of the 232 patients screened with the LFES-SQ questionnaire; only 96 patients (41.4%) accepted to attend the consultation. The diagnosis of FM was only confirmed in 20 cases. In practice, failing a specific biological marker for diagnosing FM, diagnosis is based on the ACR 1990 FM classification which recommend, in addition to chronic pain, to screen in the clinical examination for 11 out of 18 pain points according to a well-known topography [1]. Unlike pain for which the characteristics are well described and recognized, the validity of the latter criterion is debatable [21-23]. Moreover, this classification does not account for a large number of frequent pathognomonic symptoms in this disease, such as headaches, fatigue at the slightest exertion, tingling sensations in the body and extreme cold intolerance at the extremities. Nevertheless, the ACR classification continues to be used routinely by rheumatologists. A survey conducted in 2003 on 1130 general practitioners and 430 rheumatologists in France demonstrated that only 17.7% of general practitioners knew or used the ACR criteria as opposed to 83.7% of rheumatologists [24]. In this way, independently of the ACR criteria, new tools such as the regional pain score are developed to assist diagnosis [25,26]. This tool is based on the patient's self-assessment of his/her pain using a questionnaire with scores ranging from 0 to 19. Katz et al [27], in a correlation study between the clinical examination, regional pain score and screening for 11 out of 18 pain points on a population of 206 patients demonstrated the existence of a moderate agreement between these three criteria; however, the likelihood of diagnosis of FM was higher in patients with a high regional pain score. The author thus concludes that a regional pain score ≥ 8 associated with a fatigue score ≥ 6 on the VAS scale could represent a valid criterion for fibromyalgia diagnosis. In our study, a supplementary analysis of the factors associated with FM diagnosis by the rheumatologist, independently of ACR criteria, demonstrated a significant correlation between the FM diagnosis and the regional pain score. Indeed, a regional pain score greater than 10 was predictive of confirmed diagnosis by the rheumatologist with a 75% sensitivity and a 67% specificity. Similarly, the study demonstrated that other factors in addition to the regional pain score were associated with an increase in FM diagnosis. It would be worth taking these criteria into account to increase the chances of diagnosing the disease. This particularly applies to high stress levels, the quality of life and the presence of co-morbidities such as digestive disorders.

## Conclusions

Using a dual method consisting of telephone screening and clinical confirmation of the diagnosis of FM based on ACR criteria, this study showed an overall prevalence of FM of 1.6% (1.2%; 2.0%) in the French general population. The analysis of the patient questionnaires demonstrated that, independently of ACR criteria, other criteria such as a high level of stress, low SF36 scores, the presence of digestive disorders and a high regional pain score could represent useful diagnostic tools in future epidemiological studies on the prevalence of FM.

## Competing interests

Authors have received a grant from Pfizer for the participation to the study.

## Authors' contributions

SP and PR are the main authors of this paper. They contributed equally to the conception and design of the study, to the data analyses, and to the drafting of the manuscript. PR coordinated the study, and SP established the final version of the manuscript. EV contributed to the conception and design, especially the statistics, participated to the data analysis, to the draft manuscript and approved the final version. DS contributed to the overall design, revised the manuscript and approved the final version.

All authors read and have given final approval of the final manuscript.

## Pre-publication history

The pre-publication history for this paper can be accessed here:

http://www.biomedcentral.com/1471-2474/12/224/prepub
